# The transcriptional coactivator CmMBF1c is required for waterlogging tolerance in *Chrysanthemum morifolium*

**DOI:** 10.1093/hr/uhac215

**Published:** 2022-09-21

**Authors:** Nan Zhao, Chuanwei Li, Yajun Yan, Haibin Wang, Likai Wang, Jiafu Jiang, Sumei Chen, Fadi Chen

**Affiliations:** State Key Laboratory of Crop Genetics and Germplasm Enhancement, Key Laboratory of Landscaping, Ministry of Agriculture and Rural Affairs, Key Laboratory of Biology of Ornamental Plants in East China, National Forestry and Grassland Administration, College of Horticulture, Nanjing Agricultural University, 210095 Nanjing, China; State Key Laboratory of Crop Genetics and Germplasm Enhancement, Key Laboratory of Landscaping, Ministry of Agriculture and Rural Affairs, Key Laboratory of Biology of Ornamental Plants in East China, National Forestry and Grassland Administration, College of Horticulture, Nanjing Agricultural University, 210095 Nanjing, China; State Key Laboratory of Crop Genetics and Germplasm Enhancement, Key Laboratory of Landscaping, Ministry of Agriculture and Rural Affairs, Key Laboratory of Biology of Ornamental Plants in East China, National Forestry and Grassland Administration, College of Horticulture, Nanjing Agricultural University, 210095 Nanjing, China; State Key Laboratory of Crop Genetics and Germplasm Enhancement, Key Laboratory of Landscaping, Ministry of Agriculture and Rural Affairs, Key Laboratory of Biology of Ornamental Plants in East China, National Forestry and Grassland Administration, College of Horticulture, Nanjing Agricultural University, 210095 Nanjing, China; State Key Laboratory of Crop Genetics and Germplasm Enhancement, Key Laboratory of Landscaping, Ministry of Agriculture and Rural Affairs, Key Laboratory of Biology of Ornamental Plants in East China, National Forestry and Grassland Administration, College of Horticulture, Nanjing Agricultural University, 210095 Nanjing, China; State Key Laboratory of Crop Genetics and Germplasm Enhancement, Key Laboratory of Landscaping, Ministry of Agriculture and Rural Affairs, Key Laboratory of Biology of Ornamental Plants in East China, National Forestry and Grassland Administration, College of Horticulture, Nanjing Agricultural University, 210095 Nanjing, China; State Key Laboratory of Crop Genetics and Germplasm Enhancement, Key Laboratory of Landscaping, Ministry of Agriculture and Rural Affairs, Key Laboratory of Biology of Ornamental Plants in East China, National Forestry and Grassland Administration, College of Horticulture, Nanjing Agricultural University, 210095 Nanjing, China; State Key Laboratory of Crop Genetics and Germplasm Enhancement, Key Laboratory of Landscaping, Ministry of Agriculture and Rural Affairs, Key Laboratory of Biology of Ornamental Plants in East China, National Forestry and Grassland Administration, College of Horticulture, Nanjing Agricultural University, 210095 Nanjing, China

## Abstract

Waterlogging is one of the most serious abiotic stressors affecting *Chrysanthemum morifolium* during its lifespan. However, the molecular mechanisms underlying the waterlogging tolerance of chrysanthemum remain unclear. In this study, we discovered that the transcriptional coactivator MULTIPROTEIN BRIDGING FACTOR 1c (*CmMBF1c*) was significantly induced by waterlogging stress in chrysanthemums. Promoter sequence analysis and transient dual-luciferase assay using chrysanthemum protoplasts showed that the waterlogging-tolerant cultivar ‘Nannongxuefeng’ carried more response elements involved in waterlogging and hypoxia stress compared with the waterlogging-sensitive cultivar ‘Qinglu’, conferring on ‘Nannongxuefeng’ a stronger hypoxia responsive activity and higher *CmMBF1c* expression under waterlogging conditions. Subcellular localization and transcriptional activity assays showed that CmMBF1c protein was localized to the nucleus and had no transcriptional activation activity. Overexpression of *CmMBF1c* in ‘Qinglu’ enhanced its waterlogging tolerance by promoting its reactive oxygen species (ROS) scavenging ability and maintaining low ROS levels. However, RNAi-mediated knockdown of *CmMBF1c* in cultivar ‘Nannongxuefeng’ resulted in the opposite tendency. Yeast two-hybrid screening and tobacco bimolecular fluorescence complementation assays revealed that CmHRE2, a pivotal regulator of hypoxia response, could interact with CmMBF1c. In summary, this study demonstrates that CmMBF1c improves chrysanthemum waterlogging tolerance by regulating its ROS signaling pathway and interacting with CmHRE2. These findings together offer, to our knowledge, new mechanistic insights into chrysanthemum waterlogging tolerance and provide a rational foundation for future research on the genetic improvement of horticultural crops for waterlogging stress tolerance.

## Introduction

Water is among the most vital environmental factors affecting plant growth, development, morphology, and physiological and biochemical metabolism [1]. Climatic variability, in terms of events such as typhoons and long-term rainstorms, causes the flooding of rivers and rising of sea levels, resulting in excessive soil moisture and frequent waterlogging. Waterlogging dramatically reduces the oxygen (O_2_) diffusion rate in soils, which triggers O_2_ depletion, leading to the excessive accumulation of reactive oxygen species (ROS) in plants, including superoxide anions (O_2_^∙−^), hydroxyl radicals (^•^OH), hydrogen peroxide (H_2_O_2_), and singlet oxygen (^1^O_2_) [2–4]. The excessive ROS cannot be removed quickly, resulting in the peroxidation of nucleic acids, proteins, and lipid membranes in the cell, which disrupts the structure and function of the cell [2, 3]. Finally, the excessive ROS content can directly cause plant growth inhibition and yield decline and even plant death in severe cases [5, 6]. Therefore, reducing the effects of ROS under waterlogging conditions is essential for improving waterlogging tolerance in plants [7, 8].

Chrysanthemum is among the most popular ornamental plants in the world [9]. Most chrysanthemum cultivars are susceptible to hypoxia and waterlogging conditions. Owing to the susceptibility of wide areas to waterlogging damage and the seasonal concentrated rainfall in different areas, even short-term waterlogging can lead to large-scale death of chrysanthemums [10]. Therefore, waterlogging has become one of the most severe limiting factors in the industrial production of chrysanthemums. It is critical to study the molecular mechanisms of chrysanthemum waterlogging tolerance to alleviate this challenge and develop new waterlogging-tolerant cultivars. Because of the complex genetic background of chrysanthemums and the lack of genomic information, current studies on the waterlogging tolerance of chrysanthemums have mostly focused on the evaluation of the waterlogging tolerance of different chrysanthemum species and the effect of waterlogging stress on their physiology and biochemistry [10, 11]. Though several waterlogging-related genes have been cloned and investigated [12–14], the vital factors regulating waterlogging tolerance in chrysanthemums and their molecular regulatory mechanisms are still unidentified.

Transcriptional coactivators are crucial for eukaryotic gene expression because they bridge transcription factors and regulatory elements [15, 16]. Multiprotein bridging factor 1 (MBF1), a transcriptional coactivator, is widely distributed in animals, plants, and microorganisms [17]. Previous reports have shown that MBF1s are crucial for plant growth and stress responses [18–22]. *MBF1a* and *MBF1b* in *Arabidopsis* are expressed in specific tissues and can be induced by various abiotic stresses [19]. *MBF1c* is upregulated by a variety of abiotic stressors and hormone signals such as salt, drought, salicylic acid, and abscisic acid [19, 20]. *AtMBF1c* participates in the heat shock response networks, including the ethylene, salicylic acid, and trehalose signal transduction pathways [22]. Overexpression of *AtMBF1c* improves heat tolerance through ethylene signaling pathways in *Arabidopsis* [22]. In addition to heat shock stress, drought conditions and exposure to H_2_O_2_ significantly induce *TaMBF1* in wheat [23]. Overexpression of *TaMBF1* results in an enhanced tolerance to heat stress in rice [23]. In a previous study, we compared the expression of genes in waterlogging-tolerant chrysanthemum cultivar ‘Nannongxuefeng’ and waterlogging-sensitive cultivar ‘Qinglu’ under waterlogging and reoxygenation conditions using RNA-seq [24]. The results showed that *CmMBF1c* was significantly induced by waterlogging conditions and was differentially expressed between the two cultivars, indicating that *CmMBF1c* may be involved in regulating the waterlogging tolerance of chrysanthemums [24]. However, the precise functions of *CmMBF1c* under waterlogging and recovery conditions and the underlying mechanisms are yet to be determined.

In this study, to determine the role of *CmMBF1c* in chrysanthemum waterlogging responses, we cloned this gene and confirmed the dynamic expression of *CmMBF1c* in response to waterlogging stress. Overexpression of *CmMBF1c* in the waterlogging-sensitive cultivar ‘Qinglu’ led to improved waterlogging tolerance via upregulation of ROS-scavenging gene expression and reduction in ROS accumulation. On the other hand, inhibition of *CmMBF1c* in the waterlogging-tolerant cultivar ‘Nannongxuefeng’ decreased waterlogging tolerance, meditated by downregulation of the expression of ROS scavenging genes and elevation of ROS accumulation. Using yeast two-hybrid (Y2H) screening and bimolecular fluorescence complementation (BiFC) assays, we demonstrated that CmMBF1c regulates waterlogging tolerance by interacting with CmHRE2, one of the VII ERF transcription factors that regulate the hypoxic response. Overall, this study improves our knowledge of the theoretical basis of MBF1c and VII ERF transcription factor-meditated regulation of plant waterlogging tolerance and clarifies the novel molecular pathway by which CmMBF1c regulates waterlogging tolerance in chrysanthemums, which has important practical significance for enriching the molecular mechanisms of plant waterlogging tolerance and creating new waterlogging-tolerant germplasms in chrysanthemums.

## Results

### Sequence and phylogenetic analysis of CmMBF1c

**Figure 1:**
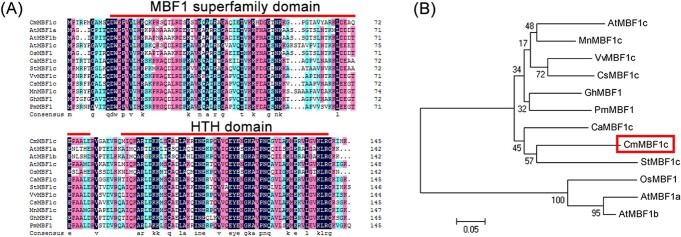
Sequence analysis of CmMBF1c and other MBF1 proteins. (A) Amino acid comparison between CmMBF1c and MBF1 homologs from other species. In the color scheme of multiple sequence alignment, navy blue represents 100% identity, pink represents 75% identity, and light blue represents 50% identity. (B) Phylogenetic tree of CmMBF1c and other MBF1 proteins of other species. Sequence details are as follows: AtMBF1c (*Arabidopsis thaliana* MBF1c, NP_565981.1), MnMBF1c (*Morus notabilis* MBF1c, XP_010088841.1), VvMBF1c (*Vitis vinifera* MBF1c, XP_002284605.1), CsMBF1c (*Cucumis sativus* MBF1c, XP_004139340.1), GhMBF1c (*Gossypium hirsutum* MBF1c, AFN70435.1), PmMBF1c (*Prunus mume* MBF1c, XP_008242608.1), CaMBF1c (*Cicer arietinum* MBF1c, XP_004512966.1), StMBF1c (*Solanum tuberosum* MBF1c, XP_006351823.1), OsMBF1 (*Oryza sativa* MBF1, ADX60234.1), AtMBF1a (*A. thaliana* MBF1a, NP_565981.1), AtMBF1b (*A. thaliana* MBF1b, NP_191427.1).

A multiprotein bridging factor gene, *CmMBF1c*, was identified, the open reading frame (ORF) sequences for which were identical in the two chrysanthemum cultivars. The ORF length of *CmMBF1c* was 438 bp, encoding a protein of 145 amino acids (Fig. 1A). To better understand the characteristics of the chrysanthemum CmMBF1c protein sequence, we performed a multiple sequence alignment between CmMBF1c and MBF1s from other species, including *Arabidopsis thaliana*, *Morus notabilis*, *Vitis vinifera*, *Cucumis sativus*, *Gossypium hirsutum*, *Prunus mume*, *Cicer arietinum*, *Solanum tuberosum*, and *Oryza sativa*. MBF1s are characterized by a conserved MBF1 superfamily domain and helix–turn–helix HTH domain (Fig. 1A). Both domains were detected in the CmMBF1c sequence, indicating that CmMBF1c belongs to the MBF1 family (Fig. 1A). Pairwise analysis of the full-length MBF1 protein sequences revealed relatively high identity between CmMBF1c and other MBF1s, suggesting that the amino acid sequence of the MBF1s in different species is relatively conserved.

To determine the evolutionary relationships between CmMBF1c and other MBF1s, we generated an unrooted neighbor-joining phylogenetic tree using the full-length MBF1 protein sequences (Fig. 1B). We observed that CmMBF1c clustered into the same branch as potato StMBF1c (XP_006351823.1), indicating a close genetic relationship between them (Fig. 1B).

### Expression pattern of *CmMBF1c* under waterlogging and reoxygenation conditions

**Figure 2:**
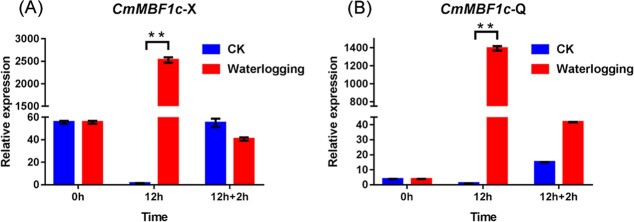
Expression of *CmMBF1c* in roots of ‘Nannongxuefeng’ and ‘Qinglu’ at 0 hours, 12 hours, and 12 hours + 2 hours recovery. (A) Expression pattern in ‘Nannongxuefeng’. (B) Expression pattern in ‘Qinglu’.

To assess the potential functions of *CmMBF1c* in the waterlogging response, we examined the expression pattern of *CmMBF1c* under waterlogging and reoxygenation conditions. *CmMBF1c* was found to be significantly induced after 12 hours of waterlogging treatment in ‘Nannongxuefeng’ and ‘Qinglu’, and recovered upon 2 hours of reoxygenation treatment after waterlogging (Fig. 2Aand B). In addition, the level of *CmMBF1c* expression was changed 2500-fold after waterlogging treatment in ‘Nannongxuefeng’, whereas only 1400-fold change was found in ‘Qinglu’ (Fig. 2A and B), suggesting that the elevation of *CmMBF1c* expression correlates positively with waterlogging tolerance in chrysanthemum.

**Table 1:** Analysis of *CmMBF1c* promoter sequences in ‘Nannongxuefeng’ and ‘Qinglu’.

Element	Number in ‘Nannongxuefeng’	Number in ‘Qinglu’	Position in ‘Nannongxuefeng’	Position in ‘Qinglu’	Function
ABRE	1		(−)138		*Cis*-acting element involved in abscisic acid responsiveness
ERE	2		(−)422, (−)1202		Ethylene-responsive element
GARE-motif	1		(+)1514		Gibberellin-responsive element
P-box		1		(−)1562	Gibberellin-responsive element
TGA-element	2		(+)23, (+)355		Auxin-responsive element
TGACG-motif		1		(+)20	*Cis*-acting regulatory element involved in Methyl Jasmonate (MeJA) responsiveness
3-AF1 binding site	2	1	(+)1188, (+)1776	(+)1230	Light-responsive element
ARE	4	2	(−)18, (−) 1559, (+)1840, (−)2488	(−)189, (−)216	*Cis*-acting regulatory element essential for anaerobic induction
Box-W1	1		(+)2239		Fungal elicitor-responsive element
CCAAT-box	2		(+)345, (−)917		MYBHv1 binding site
G-box	4	2	(+)1635, (−)2621, (−)2474, (−)2763	(−)1268, (−)1559	*Cis*-acting regulatory element involved in light responsiveness
GC-motif	1		(−)1951		Enhancer-like element involved in anoxic-specific inducibility
HSE	5	3	(+)144, (+)1173, (+)1314, (−)1780, (−)1854	(−)746, (−)1216, (−)1319	*Cis*-acting element involved in heat stress responsiveness
LTR	1		(+)106		*Cis*-acting element involved in low-temperature responsiveness
TC-rich repeats	5		(+)79, (+)1100, (−)1170, (−)1692, (+)2091		*Cis*-acting element involved in defense and stress responsiveness

### Cloning and analysis of *CmMBF1c* promoter sequences

To explore the roles of *CmMBF1c* in the waterlogging response of chrysanthemums, we cloned the *CmMBF1c* gene from ‘Nannongxuefeng’ and ‘Qinglu’. We identified promoter sequences with lengths of 2532 and 2442 bp from these two cultivars, respectively.

We performed promoter element analysis on these sequences using PlantCARE [25], which predicted both promoter sequences to be involved in the response to a variety of hormone and stress conditions (Table 1). Furthermore, the categories and distributions of the two promoter sequences showed large variation. The promoter sequence of ‘Nannongxuefeng’ *CmMBF1c* contained more response elements involved in waterlogging and hypoxia stress compared with the promoter sequence of ‘Qinglu’. These elements include the hypoxia response element ARE and the hypoxia-inducible enhancer GC motif (Table 1). It can be speculated that more hypoxic response elements may contribute to the stronger upregulation of the *CmMBF1c* gene in ‘Nannongxuefeng’ compared with that in ‘Qinglu’ in response to waterlogging stress.

**Figure 3:**
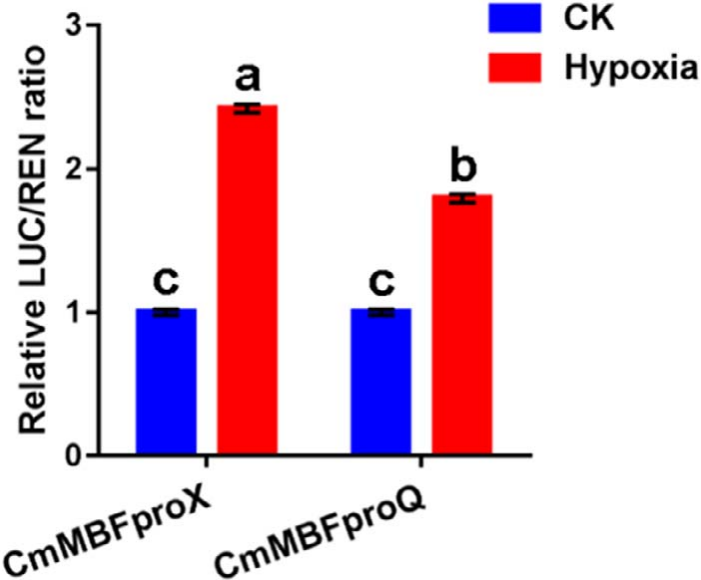
Validation of the effect of hypoxia on the activity of *CmMBF1c* promoter using dual-luciferase assay. The mean ± standard error is used to represent the values. Significant differences are indicated by letters above the columns (*P* < .05). Results represent the means of three biological replicates. CK, no hypoxia treatment.

To further investigate the potential roles of the promoter sequences in *CmMBF1c* expression regulation in the two cultivars, we constructed dual-luciferase (LUC) vectors by transforming chrysanthemum protoplasts based on two sequences. The results revealed no statistically significant difference in the relative LUC expression (LUC/REN values) when controlled by the promoter sequences from the two cultivars without treatment (CK, Fig. 3). However, under hypoxic conditions the relative expression of LUC driven by the promoter from ‘Nannongxuefeng’ was higher than that driven by the promoter from ‘Qinglu’ (Fig. 3), indicating that more hypoxic response elements in the promoter may result in stronger hypoxic responsive activity in ‘Nannongxuefeng’.

### Subcellular localization and transcription activation analysis of CmMBF1c

**Figure 4:**
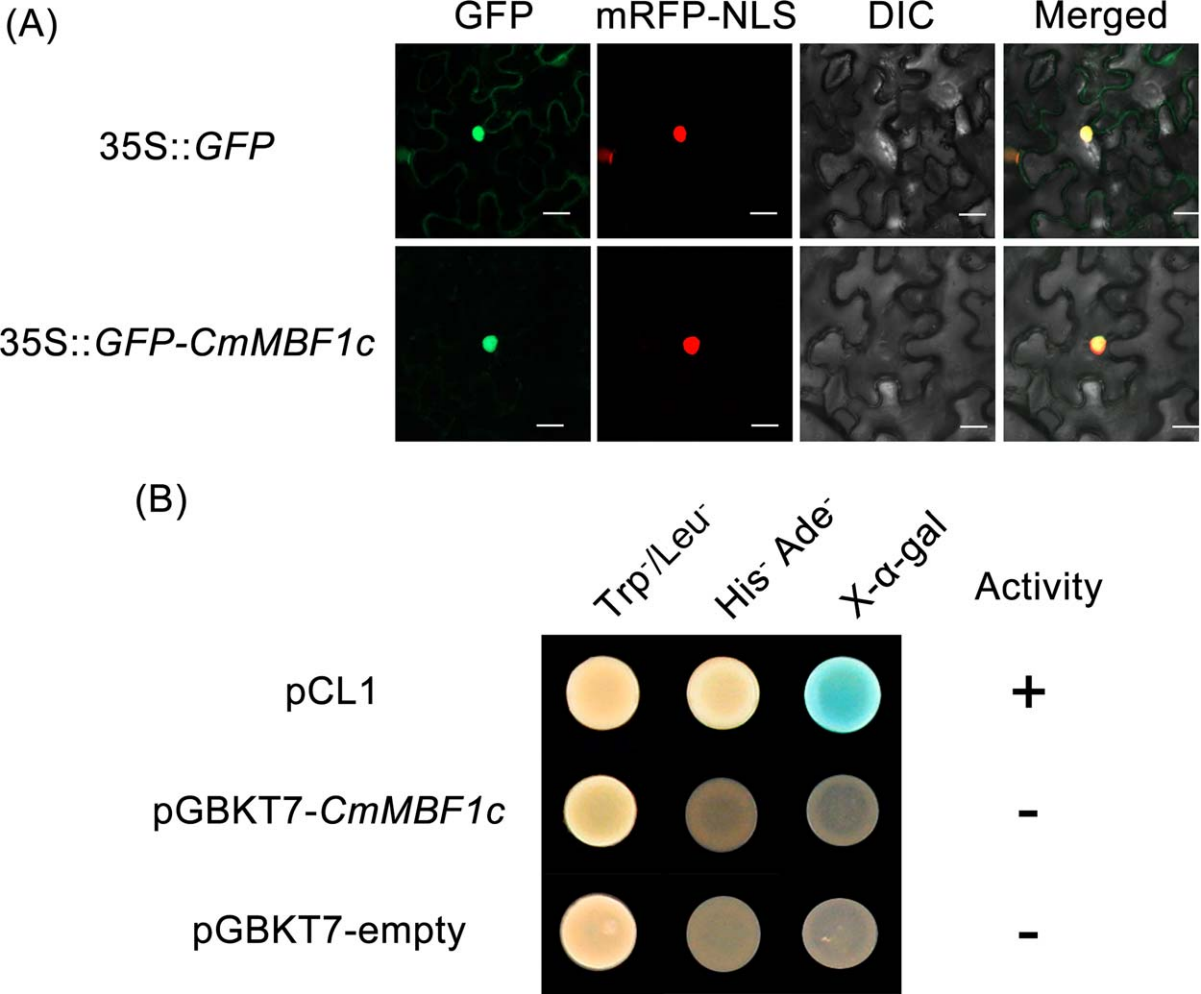
Subcellular localization and transactivation analysis of CmMBF1c. (A) Subcellular localization of CmMBF1c protein in tobacco cells. The coexpressed 35S::*D53*-*RFP* construct was used as a nuclear marker. mRFP-NLS, nuclear localization with red fluorescent protein; DIC, bright light; Merged, overlay plots. Scale bar = 20 μm (B) Analysis of transcriptional activity of CmMBF1c in yeast cells. pCL1 served as a positive control and pGBKT7 as a negative control.

To clarify the localization of CmMBF1c, we generated a CmMBF1c-GFP (GFP, green fluorescent protein) fusion vector driven by the 35S promoter. This was further introduced by *Agrobacterium* injection into tobacco cells. For tobacco cells bearing 35S::*GFP*-*CmMBF1c*, GFP was exclusively found in the nucleus (Fig. 4A), suggesting that CmMBF1c protein is localized in the nucleus.

To study whether CmMBF1c has transcriptional activation activity, we constructed a pGBKT7-*CmMBF1c* vector for yeast transformation. The yeast bearing the positive control pCL1 plasmid grew normally on SD/−Ade/−His defective medium and turned blue with exogenous application of X-α-gal (Fig. 4B), whereas neither the negative control pGBKT7-empty nor the pGBKT7-*CmMBF1c* test could grow and neither of them turned blue (Fig. 4B), indicating that CmMBF1c has no transcriptional activation activity.

### *CmMBF1c* improved waterlogging tolerance in transgenic chrysanthemums

**Figure 5:**
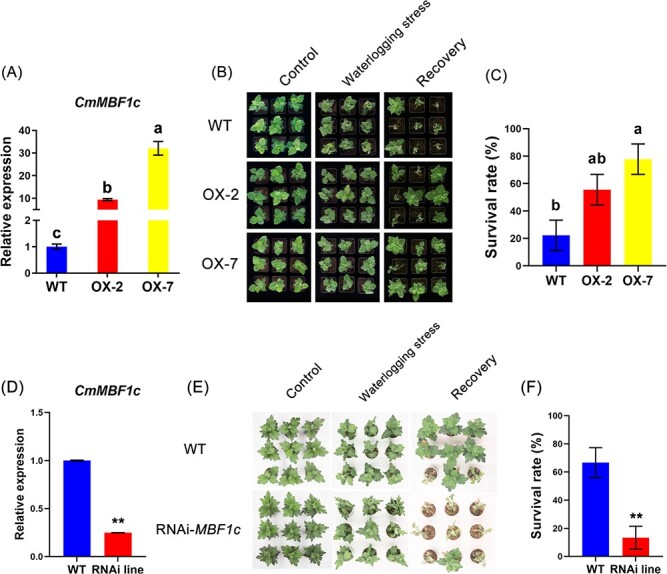
Validation of waterlogging tolerance in WT and corresponding transgenic lines. (A) Relative expression levels of *CmMBF1c* in ‘Qinglu’ and *CmMBF1c* overexpression transgenic lines. (B) Phenotypic observation of ‘Qinglu’ and *CmMBF1c* overexpression lines after waterlogging and recovery treatment. (C) Analysis of survival rate of ‘Qinglu’ and *CmMBF1c* overexpression lines after waterlogging stress. (D) Relative expression levels of *CmMBF1c* in ‘Nannongxuefeng’ and *CmMBF1c* RNAi line. (E) Phenotypic observation of ‘Nannongxuefeng’ and line after waterlogging and recovery treatment. (F) Analysis of survival rate of ‘Nannongxuefeng’ and *CmMBF1c* RNAi line after waterlogging stress. The mean ± standard error is used to represent the values. Significant differences are indicated by letters and asterisks above the columns (*P* < .01).

To further investigate the role of *CmMBF1c* in the chrysanthemum waterlogging response, *Agrobacterium* containing the overexpression vector pMDC32-*CmMBF1c* was introduced into the waterlogging-sensitive cultivar ‘Qinglu’. Positive transgenic chrysanthemums were validated by PCR (Supplementary Data Fig. S1) and two independent overexpression lines, OX-2 and OX-7, were selected for subsequent phenotypic observation and downstream gene expression analysis (Fig. 5A).

Through phenotypic observation we found that the OX-2 and OX-7 lines showed less damage than wild-type (WT) upon waterlogging treatment (Fig. 5B). After 4 days of waterlogging treatment, WT plants displayed severely wilted leaves, while leaves from the two *CmMBF1c* overexpression lines remained upright (Fig. 5B). After recovery, the two overexpression lines outperformed the WT plants in terms of growth (Fig. 5B). The survival rate of the WT plants was 22.22% (Fig. 5C). However, the survival rates of the two *CmMBF1c* overexpression lines (OX-2 and OX-7) were 55.56% and 77.78%, respectively (Fig. 5C).

Given that greater elevation of *CmMBF1c* expression was observed in the waterlogging-tolerant cultivar ‘Nannongxuefeng’ than in ‘Qinglu’ (Fig. 2), we generated *CmMBF1c* RNAi lines in ‘Nannongxuefeng’ by *Agrobacterium*-mediated transformation. Although the transformation efficiency of ‘Nannongxuefeng’ was extremely low, we obtained one *CmMBF1c* RNAi-positive transgenic line (Supplementary Data Fig. S2). Waterlogging treatment showed that the *CmMBF1c* RNAi line was more sensitive to waterlogging stress than the WT (Fig. 5E). After the recovery treatment, the survival rate of the RNAi line significantly decreased (Fig. 5F). Taken together, these results indicate that *CmMBF1c* plays a key role in chrysanthemum waterlogging tolerance.

### *CmMBF1c* altered ROS levels and ROS scavenger activities

**Figure 6:**
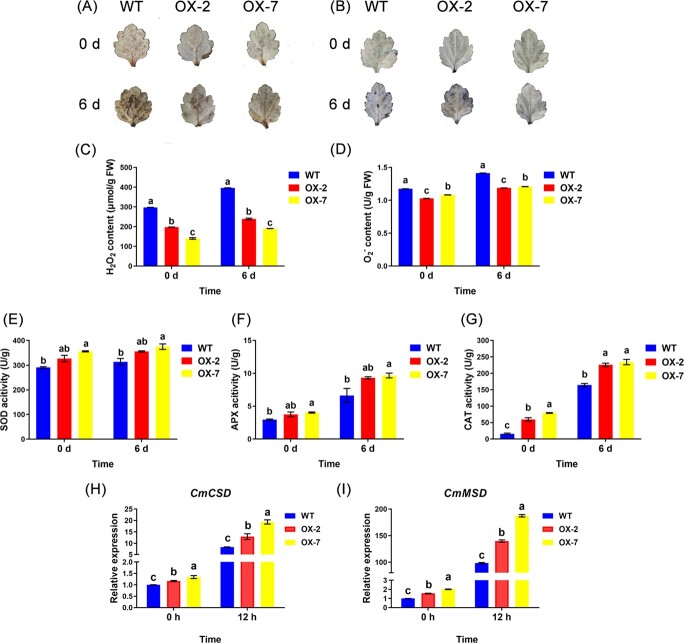
Measurement of ROS contents, ROS scavenger activities and ROS signaling-related gene expression of ‘Qinglu’ and *CmMBF1c* overexpression lines. (A) DAB staining. (B) NBT staining. (C) Leaf H_2_O_2_ content. (D) Leaf O_2_^∙−^ content. (E) Leaf SOD activity. (F) Leaf APX activity. (G) Leaf CAT activity. (H, I) expression of ROS signaling-related genes in ‘Qinglu’ WT, OX-2, and OX-7. The mean ± standard error is used to represent the values. Significant differences are indicated by letters above the columns (*P* < .05).

**Figure 7:**
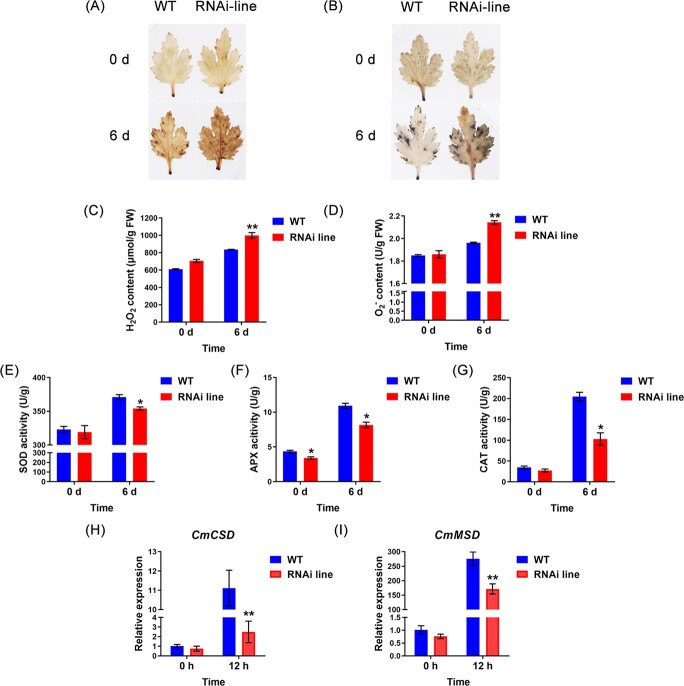
Measurement of ROS contents, ROS scavenger activities and ROS signaling-related gene expression of ‘Nannongxuefeng’ and *CmMBF1c* RNAi line. (A) DAB staining. (B) NBT staining. (C) Leaf H_2_O_2_ content. (D) Leaf O_2_^∙−^ content. (E) Leaf SOD activity. (F) Leaf APX activity. (G) Leaf CAT activity. (H, I) Expression of ROS signaling-related genes in ‘Nannongxuefeng’ WT and *CmMBF1c* RNAi line. The mean ± standard error is used to represent the values. Significant differences are indicated by asterisks above the columns (^*^P < .05, ^**^P < .01).

Waterlogging stress promotes rapid ROS accumulation in plants [26, 27]. To further elucidate the role of *CmMBF1c* in regulating ROS levels in chrysanthemum, we used diaminobenzidine (DAB) and nitroblue tetrazolium (NBT) staining to examine H_2_O_2_ and O_2_^∙−^ levels in the leaves of the WT and transgenic chrysanthemums following waterlogging treatment. Leaves from *CmMBF1c* overexpression lines clearly showed less DAB and NBT staining intensities than those from WT plants (Fig. 6A and B). Under normal or waterlogging conditions, the two overexpression lines showed conspicuously lower H_2_O_2_ and O_2_^∙−^ levels than the WT (Fig. 6C and D). However, the *CmMBF1c* RNAi line exhibited higher H_2_O_2_ and O_2_^∙−^ contents than the WT plants (Fig. 7A–D), indicating that *CmMBF1c* enhances chrysanthemum waterlogging tolerance through the ROS-mediated pathway.

To further investigate the role of *CmMBF1c* in regulating ROS homeostasis in chrysanthemums, the ROS-scavenging activities of the WT and transgenic lines were determined. The activities of superoxide dismutase (SOD), catalase (CAT), and ascorbate peroxidase (APX) in the *CmMBF1c* overexpression lines were higher than those in WT plants under both normal and waterlogging conditions (Fig. 6E–G); whereas the *CmMBF1c* RNAi line showed lower activities than WT plants (Fig. 7E–G). The results demonstrated that *CmMBF1c* maintains a low level of ROS accumulation by promoting ROS scavenging activity, leading to enhanced waterlogging tolerance.

To further investigate the roles of *CmMBF1c* in the ROS signaling pathway in chrysanthemum, we examined the expression pattern of ROS-scavenging genes in *CmMBF1c* transgenic lines and WT plants by qRT–PCR. The results demonstrated that the ROS scavenging genes *Cu/Zn-SOD* (*CmCSD*) and *Mn-SOD* (*CmMSD*) were significantly upregulated in the overexpression lines compared with WT under both normal and waterlogged conditions (Fig. 6H and I). In RNAi plants, we observed low expression of *CmCSD* and *CmMSD* under waterlogged conditions (Fig. 7H and I). These findings suggest that *CmMBF1c* improves waterlogging tolerance in chrysanthemum by decreasing ROS accumulation and enhancing ROS-scavenging gene expression.

### CmMBF1c interacts with CmHRE2 in yeast and tobacco cells

**Figure 8:**
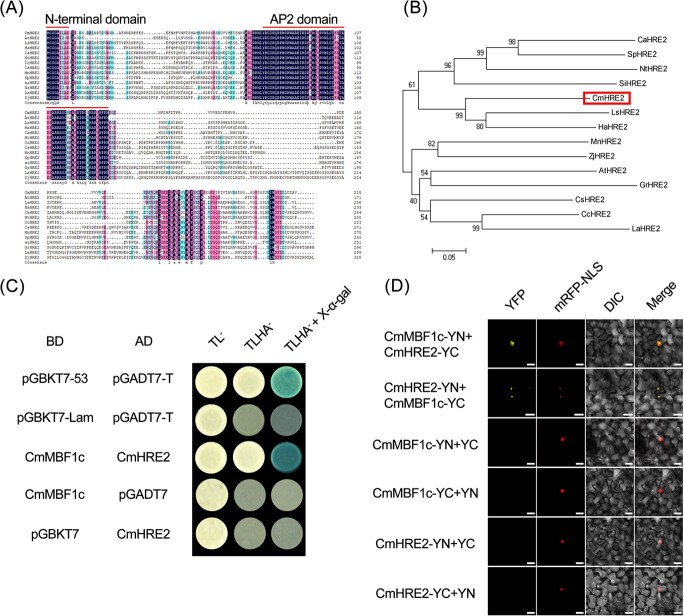
Sequence analysis of CmHRE2 and other HRE2 proteins, and interaction between CmMBF1c and CmHRE2 in yeast and tobacco cells. (A) Amino acid comparison between CmHRE2 and HRE2 homologs from other species. In the color scheme of multiple sequence alignment, navy blue represents 100% identity, pink represents 75% identity, and light blue represents 50% identity. (B) Phylogenetic tree of CmHRE2 and other HRE2 proteins of other species. The sequence details are as follows: CaHRE2 (*Capsicum annuum* HRE2, NP_001311812.1), SpHRE2 (*Solanum pennellii* HRE2, XP_015087747.1), NtHRE2 (*Nicotiana tabacum* HRE2, XP_016475776.1), SiHRE2 (*Sesamum indicum* HRE2, XP_011079624.1), LsHRE2 (*Lactuca sativa* HRE2, XP_023740943.1), HaHRE2 (*Helianthus annuus* HRE2, XP_022035166.1), MnHRE2 (*Morus notabilis* HRE2, XP_010107742.1), ZjHRE2 (*Ziziphus jujuba* HRE2, XP_015891869.1), AtHRE2 (*Arabidopsis thaliana* HRE2, NP_182274.1), GrHRE2 (*Gossypium raimondii* HRE2, XP_012473480.1), CsHRE2 (*Cucumis sativus* HRE2, XP_004152238.1), CcHRE2 (*Cajanus cajan* HRE2, KYP74231.1), LaHRE2 (*Lupinus angustifolius* HRE2, XP_019441326.1). (C) Interaction between CmMBF1c and CmHRE2 in yeast cells. (D) BiFC analysis of interaction between CmMBF1c and CmHRE2 in tobacco cells. The coexpressed 35S::*D53*-*RFP* construct was used as a nuclear marker. YFP, yellow fluorescence channel; mRFP-NLS, nuclear localization with red fluorescent protein; DIC, bright light; Merge, overlay plots. Scale bar = 20 μm.

To gain further insight into the mechanisms that control waterlogging tolerance by *CmMBF1c*, we screened the chrysanthemum Y2H library using pGBKT7-CmMBF1c to identify the proteins that potentially interact with CmMBF1c. A protein named CmHRE2 was isolated from this screen. The full-length *CmHRE2* ORF was 684 bp and encoded 228 amino acids (Fig. 8A). The N-terminus of CmHRE2 contained an MCGGAI/L motif and an AP2 domain (Fig. 8A). Phylogenetic tree analysis of CmHRE2 and HRE2 from other species showed that CmHRE2 was closely related to LsHRE2 and HaHRE2 but distantly related to CsHRE2, CcHRE2, and LaHRE2 (Fig. 8B). These results indicate that chrysanthemum CmHRE2 is a typical Group VII ERF transcription factor.

Next, we validated the interaction between CmMBF1c and CmHRE2 by using the Y2H assay (Fig. 8C). The positive control group pGBKT7–53 + pGADT7-T grew normally on SD/−Trp/−Leu/−His/−Ade yeast four-drop-out medium, and the yeast cells turned blue with additional application of X-α-gal (Fig. 8C), whereas the other three negative control groups did not grow on the four-drop-out medium, regardless of the additional application of X-α-gal (Fig. 8C). However, the combination of pGBKT7-CmMBF1c + pGADT7-CmHRE2 grew normally on the four-drop-out medium, and the yeast cells turned blue with the additional application of X-α-gal (Fig. 8C), confirming that CmMBF1c interacts with CmHRE2 in yeast cells.

Finally, we employed a BiFC assay to validate the interaction between CmMBF1c and CmHRE2 (Fig. 8D). Yellow fluorescent protein (YFP) signals were detected in both CmMBF1c-YN + CmHRE2-YC and CmHRE2-YN + CmMBF1c-YC in tobacco leaf cells (Fig. 8D), further confirming the interaction between CmMBF1c and CmHRE2.

## Discussion

Transcription coactivators play a vital role in regulating growth, development, and stress responses in eukaryotes by interacting with nuclear receptor activation regions and basic transcriptional mechanisms [28]. In animals, the transcriptional coactivator MBF1 promotes target gene expression by altering its subcellular localization from the cytoplasm to the nucleus [29]. In *Arabidopsis*, all three MBF1s are predominantly located in the nucleus [30]. In this study, CmMBF1c was similarly localized to the nucleus (Fig. 4B). In plants, the expression of *MBF1s* is induced by various stresses, which is not the case with *MBF1s* in animals. Consequently, it is possible that MBF1s regulate the expression of downstream genes in animals by nuclear re-localization; however, in plants downstream genes are regulated mainly by changes in MBF1s level [30].

Promoters play a vital role in regulating gene expression at the transcriptional level [31]. Changes in the promoter regulatory elements can result in variations in gene expression [32]. In order to explore the mechanisms underlying the difference in *CmMBF1c* at the transcriptional level between the two chrysanthemum cultivars, we cloned and analyzed the promoter sequences of *CmMBF1c* from ‘Nannongxuefeng’ and ‘Qinglu’. Results showed that the two promoter sequences are different, and the *CmMBF1c* promoter sequence from ‘Nannongxuefeng’ harbored more hypoxia-induced elements (Table 1), leading to stronger hypoxic response activity (Fig. 3), indicating that more hypoxic response elements in the promoter may result in stronger hypoxic responsive activity in ‘Nannongxuefeng’. The exact mechanism by which *CmMBF1c* is regulated in response to hypoxia requires the identification of additional upstream regulators under waterlogging conditions.

The overexpression of *MBF1c* can improve plant tolerance to multiple stresses [23, 33, 34]. *MBF1c*, a vital regulator of heat tolerance in *A. thaliana*, is dramatically induced by heat stress [22, 35]. Overexpression of *AtMBF1c* in *Arabidopsis* improves its tolerance to oxidative damage and heat stress [22, 34]. In addition, salt, drought, trehalose, salicylic acid, methyl viologen, abscisic acid, and other stresses and hormone treatments can increase the expression of *AtMBF1c* [19, 20]. *MBF1c* is induced by salicylic acid (SA), methyl jasmonate (MJ), abscisic acid, and *Ralstonia solanacearum*, and overexpression of *MBF1c* results in improved resistance to *R. solanacearum* in *S. tuberosum* [21]. However, whether *MBF1c* participates in waterlogging tolerance has not been reported. In this study, we overexpressed *CmMBF1c* in the waterlogging-sensitive chrysanthemum cultivar ‘Qinglu’, and generated *CmMBF1c* RNAi lines using the waterlogging-tolerant chrysanthemum cultivar ‘Nannongxuefeng’. Our experiments showed that *CmMBF1c* enhanced waterlogging tolerance by regulating the ROS signaling pathway in chrysanthemums (Figs 6 and 7). Thus, we have unraveled new roles of *MBF1s* in plant stress responses and new mechanistic insights into chrysanthemum waterlogging tolerance. Further exploration of whether and how other *CmMBF1s* are involved in waterlogging response will also be of interest.

MBF1 is a highly conserved cotranscription factor. Its N-terminus can bind to a variety of catalysts. When its DNA activation domain encounters an activator, MBF1 interacts with transcription factors to induce a change from its unfolded state to form a folded state [36]. The middle part of the C-terminal is a protein–protein interaction region [36], while the C-terminal has an HTH domain, which can form a dimer through HTH, and MBF1 also interacts with other genes [37]. For example, the interaction between AtMBF1c and TPS5 in *Arabidopsis* is required for the heat shock response [22, 38]. The A20/AN1 zinc finger protein AtSAP5 interacts with AtMBF1c to regulate heat shock-responsive gene expression [39]. In this study, using Y2H screening and BiFC validation, we found that CmMBF1c interacted with CmHRE2, a VII ERF transcription factor (Fig. 8). It is possible that CmMBF1c regulates downstream genes involved in the chrysanthemum waterlogging response by binding to CmHRE2. Further research to verify the potential mechanism of engineered transgenes will be conducted in a future study.

*Arabidopsis* VII ERFs regulate the opening and closing of hypoxic responses through the N-end rule pathway [40, 41]. HRE2, a key member of the VII ERF transcription factor family, plays an important role in mediating plant waterlogging tolerance [42–44]. *HRE2* is significantly induced by hypoxia conditions, and overexpression of *HRE2* significantly enhances the tolerance of hypoxia in *Arabidopsis* [43, 45]. As a key regulator of waterlogging tolerance, HRE2 partners required for waterlogging regulation have not yet been investigated. Our study revealed, for the first time, that CmHRE2 interacts with CmMBF1c, a co-transcription factor, contributing to hypoxia and the waterlogging responses. Further genetic validation is critical to uncover the mechanistic details of the CmMBF1c-CmHRE2-mediated waterlogging response in plants.

## Materials and methods

### Plant materials

The chrysanthemum cultivars ‘Qinglu’ and ‘Nannongxuefeng’ were obtained from the Chrysanthemum Germplasm Resource Preserving Centre of Nanjing Agricultural University (Nanjing, Jiangsu, China). Seedlings of similar size at the six- to eight-leaf stage were planted and cultivated in a culture room at 22 ± 1°C with a photoperiod of 16 hours light/8 hours dark.

### Isolation and sequence analysis of *CmMBF1c*

Gene-specific primers CmMBF1c-F and CmMBF1c-R were designed to amplify the complete *CmMBF1c* cDNA sequence (Supplementary Data Table S1). Amino acid comparison between CmMBF1c and MBF1 homologs from other species was performed by alignment using DNAMAN 9.0.1 (Lynnon Biosoft, Canada). The algorithm in DNAMAN software first produces a homology matrix based on the sequence variability among molecular identities and then applies a correction method before aligning all sequences progressively [46]. The dynamic alignment method is used with analytical parameters set at 10 for gap open penalty, 1 for gap extension penalty, and 30% for delay divergent sequences. Bootstrap values were obtained upon 1000 trials. Phylogenetic analysis of MBF1s was based on the neighbor-joining method using MEGA 7.0 [47].

### Subcellular localization of CmMBF1c

The p35S::GFP-*CmMBF1c* fusion vector was generated for detection of the subcellular localization of CmMBF1c. The *CmMBF1c* sequence was amplified by PCR using primers CmMBF1c-1A-F and CmMBF1c-1A-R harboring the KpnI and XhoI sites (Supplementary Data Table S1). The entry vector pENTR™1A and the CmMBF1c amplified fragment were digested with KpnI and XhoI, separately. Then the corresponding fragments were recycled and ligated to generate pENTR™1A-*CmMBF1c*. The LR reaction method was used for recombination to generate pMDC43-*CmMBF1c*. Both the 35S::*GFP*-*CmMBF1c* and the 35S::*GFP* plasmids were transiently transformed into tobacco leaves by *Agrobacterium* injection. The coexpressed 35S::*D53*-*RFP* construct was used as a nuclear marker [48]. The fluorescence signal was detected using a confocal laser scanning microscope (LSM780, Zeiss, Germany).

### Transcriptional activity analysis of *CmMBF1c*

The constructed and correctly sequenced pENTR1A-*CmMBF1c* plasmid was digested with PvuI (NEB, USA) single enzyme, and the linear fragment recovered from the gel was recombined with pGBKT7 empty vector after laboratory modification, and then transformed into *Escherichia coli* competent *DH5α*, which was coated on LB/Kan^+^ resistant medium for overnight culture. On the second day, the clones were selected for bacterial fluid testing, and the positive bacterial fluid was selected for testing. The correct plasmids were selected using DNAMAN 9.0.1 software (Lynnon Biosoft, Canada).

A yeast transformation kit (Matchmaker™ Yeast Transformation System 2, Clontech, USA) was used for transcriptional activity analysis of *CmMBF1c*. pGBKT7-*CmMBF1c*, negative control pGBKT7 and positive control pCL1 were transformed into yeast Y2H competent cells separately, and coated on the corresponding defective solid medium. The bacterial solutions of negative control pGBKT7 and pGBKT7-*CmMBF1c* were coated on SD/−Trp medium, and yeast solution transformed into positive control pCL1 was coated on SD/−Leu medium. The cells were cultured at 30°C for 3 days, and the monoclones were selected and cultured in SD/−Ade/−His + X-α-gal medium. The growth status of monoclones was observed continuously and then photographed.

### Cloning and elemental analysis of *CmMBF1c* promoter

DNA of ‘Qinglu’ and ‘Nannongxuefeng’ chrysanthemum was extracted by the CTAB method [49]. The promoter of *CmMBF1c* in the two varieties was amplified using a Genome Walking Kit (Takara, Japan). Elemental analysis of the *CmMBF1c* promoter was predicted using PlantCARE [25].

### Transient transformation of chrysanthemum protoplasts

The *CmMBF1c* promoter sequences from ‘Nannongxuefeng’ and ‘Qinglu’ were amplified using the primers pGreen-proX-F/pGreen-proX-R (Supplementary Data Table S1) and pGreen-proQ-F/pGreen-proQ-R (Supplementary Data Table S1) with PstI and BamHI and were inserted into the pGreenII-0800-LUC vector [50]. Transient transformation of chrysanthemum protoplasts was performed as previously described [51]. Hypoxia treatment in chrysanthemum protoplasts was performed as previously described [52]. The protoplasts were collected after treatment and lysis was performed using the Dual-Luciferase^®^ Reporter Assay System (Promega, Madison, WI, USA). The relative LUC and REN activity was measured with a GloMax^®^20/20 (Promega, Madison, WI, USA). Three biological replicates were included for each condition.

### Genetic transformation of CmMBF1c in chrysanthemum and qRT–PCR analysis

The overexpression vector pMDC32-*CmMBF1c* and RNAi-*CmMBF1c* plasmids were transformed into the *Agrobacterium tumefaciens* EHA105 strain for transformation of chrysanthemum, which was performed as previously described [53]. The Multisource Genomic DNA Miniprep Kit (Axygen, USA) was used to extract DNA from putative transgenic chrysanthemum lines and WT plants. A PCR method was used to detect the *CmMBF1c* transgenic lines, with the primer pair Hyg-F/R (Supplementary Data Table S1).

To analyze the expression of *CmMBF1c* in transgenic lines, RNA of transgenic and WT was extracted and reverse-transcribed, using a Quick RNA Isolation Kit (Waryong, Beijing, China) and the reverse transcription M-MLV kit (TaKaRa, Tokyo, Japan), respectively. The expression of *CmMBF1c* was detected by qRT–PCR using the primer pair CmMBF1c-RT-F/R (Supplementary Data Table S1), as previously described [24]. Three biological replicates were performed for the calculation of expression level. As a reference gene, the chrysanthemum *EF1α* gene (GenBank accession number KF305681) was utilized, and the matching primer pair CmEF1α-F/R is given in Supplementary Data Table S1.

Roots were collected at 0 and 12 hours following waterlogging treatment to assess the expression levels of ROS scavenger genes *CmCSD* and *CmMSD*. As previously described, the identical procedure was employed for RNA extraction and qRT–PCR, and three biological replicates were performed [24]. Supplementary Data Table S1 contains the sequences of all relevant primers.

### Identification of waterlogging tolerance in chrysanthemum

The *CmMBF1c* overexpression lines, *CmMBF1c* RNAi line and WT chrysanthemums were treated with waterlogging for 4 days at the six- to eight-leaf stage for identification of waterlogging tolerance. The waterlogging treatments in chrysanthemum were performed as previously described [24]. The pots of chrysanthemums were placed in a 28 cm × 14 cm × 14 cm container filled with tap water, and the water level in the pots was maintained at ~3 cm above the soil surface. Then plants were relieved from waterlogging conditions 30 days of reoxygenation recovery. Control plants remained well-watered throughout the experiment. The treatments were repeated three times, using nine plants in each replication to calculate the survival rate of the transgenic and WT chrysanthemums.

### Measurement of physiological indicators of waterlogging tolerance for chrysanthemum

DAB and NBT staining were used to exhibit the accumulation of H_2_O_2_ and O_2_^∙−^ in the transgenic chrysanthemum plants as previously described [53, 54]. A hydrogen peroxide assay kit (no. A064-1-1) and superoxide anion assay kit (no. A052–1-1) provided by Nanjing Jiancheng Bioengineering Institute (Jiangsu Province, China) were used for H_2_O_2_ and O_2_^∙−^ quantification respectively, following the manufacturer’s instructions. Three biological replicates were included for each condition.

The activities of SOD, CAT and APX of chrysanthemum were quantified using a superoxide dismutase assay kit (no. A001-1-2), catalase assay kit (no. A007-1-1) and ascorbate peroxidase test kit (no. A123-1-1), respectively, provided by Nanjing Jiancheng Bioengineering Institute (Jiangsu Province, China). Three biological replicates were included for each condition.

### Yeast two-hybrid screening assay

The pGBKT7-*CmMBF1c* vector was transformed into Y2H yeast cells as a bait for Y2H screening. Roots of chrysanthemum cultivar ‘Nannongxuefeng’ were collected at 0 and 12 hours of waterlogging treatment and at 12 hours + 2 hours of recovery for the construction of the cDNA library. The cDNA library was used to perform the Y2H screening assay and positive clones were further confirmed by sequencing. The full-length ORF sequence of *CmHRE2* was amplified and inserted into the pGADT7 vector for Y2H assay detection, using the primer pair CmHRE2 AD-F/R (Supplementary Data Table S1) with KpnI and XhoI, respectively. A yeast transformation kit (Matchmarker™ Yeast Transformation System 2, Clontech, USA) was used for Y2H assay detection, and three groups – pGBKT7-*CmMBF1c +* pGADT7-*CmHRE2*, pGBKT7-*CmMBF1c +* pGADT7, and pGBKT7 *+* pGADT7-*CmHRE2* – and the negative control group pGBKT7-Lam + pGADT7-T and the positive control group pGBKT7-53 + pGADT7-T were transformed into yeast Y2H competent cells separately, and coated on SD/−Trp/−Leu medium and SD/−Trp/−Leu/–His/−Ade medium. The cells were cultured at 30°C for 3 days, and the monoclones were selected and cultured in SD/−Trp/−Leu/–His/−Ade + X-α-gal medium. The growth status of monoclones was observed continuously and then photographed. All detailed procedures were performed following the manufacturer’s instructions (Clontech).

### Bimolecular fluorescence complementation assay

The *CmMBF1c* and *CmHRE2* sequences were amplified and inserted into the pSPYNE/YCE vector for YFP signal detection [55], using the primer pairs CmMBF1c YFP-F/R (Supplementary Data Table S1) and CmHRE2 YFP-F/R (Supplementary Data Table S1) with XbaI and KpnI, respectively. The BiFC assay between CmMBF1c and CmHRE2 proteins was performed as previously described [56]. YFP signal was detected using a confocal laser scanning microscope (LSM780, Zeiss, Germany) [57].

### Statistical analysis

Statistical significance was determined using SPSS 23.0 (SPSS Inc., Chicago, IL, USA), based on the one-way ANOVA method. Duncan’s test was performed to determine the differences between treatments.

## Acknowledgements

This work was financially supported grants from National Natural Science Foundation of China (31730081, 31870306), the National Key Research and Development Program of China (2019YFD1001500, 2018YFD1000402), the earmarked fund for Jiangsu Agricultural Industry Technology System, and a project funded by the Priority Academic Program Development of Jiangsu Higher Education Institutions.

## Author contributions

F.D.C., N.Z., and J.F.J. designed the experiments. N.Z., C.W.L., Y.J.Y., and L.K.W. performed the experiments. F.D.C., N.Z., L.K.W., H.B.W., J.F.J., and S.M.C. analyzed the data and wrote the manuscript. N.Z., L.K.W., and C.W.L. revised the manuscript. All authors read and approved the final manuscript.

## Data availability

All data generated or analyzed during this study are included in this article.

## Conflict of interest

The authors declare no conflict of interest.

## Supplementary data

Supplementary data is available at *Horticulture Research* online.

## Supplementary Material

Web_Material_uhac215
