# Supplementary figures and images for: The transcriptional coactivator CmMBF1c is required for waterlogging tolerance in Chrysanthemum morifolium
Source: Hortic Res. 2022 Sep 21;9:uhac215. doi: 10.1093/hr/uhac215 (PMC9720447; doi:10.1093/hr/uhac215)

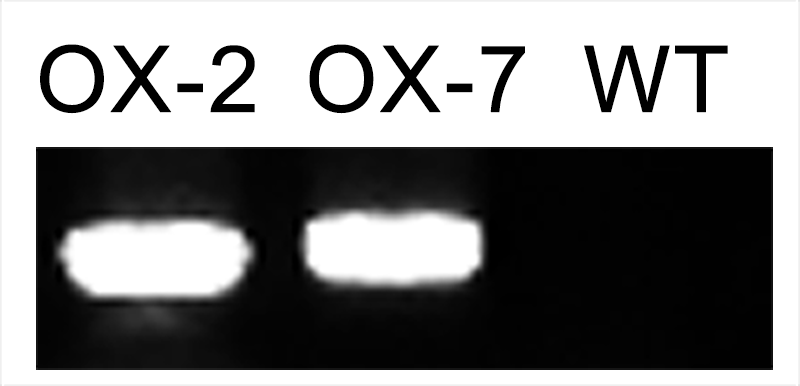

Supplement: Web_Material_uhac215 [file web_material_uhac215.zip › Figure S1.tif]

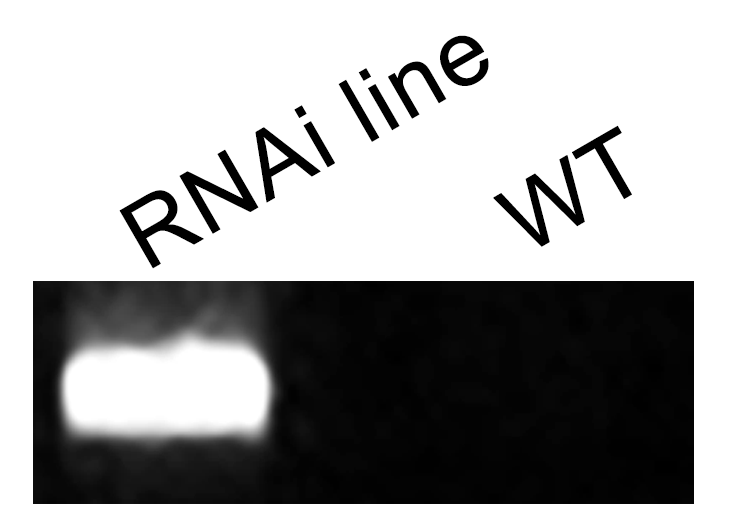

Supplement: Web_Material_uhac215 [file web_material_uhac215.zip › Figure S2.tif]
